# A retrospective case series of the surgical management of thirty-one penile fibropapilloma cases presented to University College Dublin Veterinary Hospital (UCDVH) between 2017 and 2023

**DOI:** 10.1186/s13620-024-00270-3

**Published:** 2024-04-30

**Authors:** Eilidh Elizabeth Thomson, Emmet Thomas Kelly, Marijke Eileen Beltman, Eoin Gerard Ryan

**Affiliations:** School of Veterinary Medicine, University College Dublin, Belfield, Dublin 4, Republic of Ireland

**Keywords:** Penile fibropapilloma, Warts, Bovine papilloma virus, Bull, Surgery, Internal pudendal nerve block

## Abstract

**Background:**

Penile fibropapilloma is a condition caused by bovine papilloma virus and is frequently encountered in young bulls. Penile fibropapillomatosis is thought to be spread through homosexual mounting behaviour. Fibropapillomas of the penis are painful, often bleed and can impede normal intromission. Treatment may range from allowing time for slow, spontaneous regression to surgical resection but recurrence following surgery is reported by some authors.

**Case Presentation:**

Thirty one bulls that were presented to University College Dublin Veterinary Hospital from March 2017 to March 2023 for surgical resection of penile fibropapillomas were included in this retrospective case series. Twenty-seven of the 31 bulls (87%) were under two years of age. The majority (42%) of bulls presented were Hereford, but Angus, Charolais, Holstein-Friesian and Limousin breeds were also seen. Following examination and diagnosis of penile fibropapilloma, regional anaesthesia (xylazine-procaine epidural and internal pudendal nerve block) and standing surgical intervention (resection and cautery) was performed in each case. Phone call follow-up was performed by one author (EET) in all 31 cases and 2 cases out of the 28 that were contactable showed post-surgical recurrence of penile fibropapillomatosis (i.e., 7.1% recurrence rate).

**Conclusion:**

This case series summarises the history and presenting findings of 31 bovine penile fibropapilloma cases and describes a regional anaesthetic and standing surgical approach for successful case management.

## Background

Fibropapillomatosis in the bovine animal is caused by bovine papilloma virus (BPV) [1, 2]. BPV is a non-enveloped, icosahedral double-stranded DNA virus [3] that has a worldwide distribution [4]. There are thirteen BPV genotypes, of which subtypes 1, 2, 5 and 13 can transmit to other species including buffalo [5] and horses [6]. BPV causes benign, tumour-like lesions (commonly called warts) in the skin and mucosa [7], which can spread all over the body including the skin around the face, intestines and teats [8]. Those warts found on the penis of the bull are most commonly classified as fibropapillomas, as they have more proliferation of the connective tissue adjacent to the epithelium [9], similar to “sarcoids” in horses. Penile fibropapillomas of the bull are generally caused by BPV subtype 1 [7].

BPV can be spread by fomites such as fences which then can cause small abrasions cutaneously, allowing entry of the virus [4]. Penile fibropapillomatosis is also commonly seen when bulls are kept in close contact and are mounting each other [1, 4, 10]; often several bulls in a group will develop warts [11]. BPV appears to stimulate the basal cells of the epithelium, causing some to degenerate and others to proliferate resulting in excessive growth and wart formation [4]. These lesions can progress to neoplasia during times of immunosuppression [7].

Abnormalities of bulls intended for breeding are common, with a bull breeding soundness examination (BBSE) failure rate of 19–28% reported [12]. Using the Society for Theriogenology classification, most failures are due to abnormal sperm morphology but physical abnormalities can also be a cause of failure [12]. Penile fibropapillomas are a common physical abnormality, with one abattoir survey of 550 bulls in Northern Australia showing that penile fibropapillomatosis was present in 0.18% of bulls examined (age range: 9 months to > 7 years old) [13]. When Spitzer et al., (1988) [14] examined 862 yearling bulls for BBSE, they found that 109 (12.6%) failed. Of the bulls that failed; persistent frenulum (16.5%) was the most common reason for failure, followed by penile fibropapillomatosis (1.8%). Therefore, management of penile fibropapillomatosis is necessary in a number of bulls that fail to pass a BBSE due to this abnormality.

BPV-1 mainly affects young bulls (i.e., those less than two years old) [1, 4, 10]. The majority of the lesions are narrow-based and found at the tip of the penis [4]. Often, they are not seen until they are large and the bulls are unable to retract the penis into the prepuce [4]. The most common treatment options are allowing time for spontaneous regression [7], surgery, and the administration of an autogenous vaccine [1, 10]. However, cure rates associated with the use of autogenous vaccines have generally been found to be poor [4] and few cases documenting the use of autogenous vaccines for the treatment of penile fibropapillomatosis are reported. Recurrence rate following surgery has been described to be approximately 10% [15] but literature in this area is scant. There are two reports on the use of anthelmintics for treatment of cutaneous papillomatosis [16, 17], however, none specifically referring to penile fibropapillomatosis.

The objectives of this case series were to summarise the findings and outcomes of penile fibropapillomatosis cases admitted to University College Dublin Veterinary Hospital (UCDVH) for surgical resection during the period of March 2017 to March 2023. The anaesthetic and surgical management specific to the UCDVH are also described below.

## Case presentation

### Medical record review

Electronic (Vetscope) medical records from UCDVH were searched to identify bulls that underwent surgery for the removal of penile fibropapillomas between March 2017 and March 2023. Search terms used in the electronic records included: “penis”, “penile”, “warts” and “fibropapilloma”. Additionally, any photos that were recorded at the time of the procedure for teaching purposes, were retrieved from the clinician/s in charge. Information available on the bulls included: individual identification number, herd number, breed and medications administered at the hospital. Further information collected by the author included: date of birth, pedigree status and owner contact details. Follow-up was performed via telephone contact by a single author (EET).

### Patient Signalment

A total of 31 cases were referred to UCDVH for surgical resection of penile fibropapillomas during the study period. Of this cohort, 87% of the bulls were under 2 years of age with an average age of 19 months old (range: 14.3–27.8 months old). All bulls were pedigree and intended for use as breeding bulls. A range of breeds were examined; 42% of the cases seen were in Hereford bulls, 23% in Angus, 16% in Limousin, 13% in Holstein-Friesian and 6% in Charolais. Most farms referred one or two cases over the study period; however, there were three (out of 15 farms) that presented more than two animals in the space of six years. Of these, some were seen on the same day but otherwise there was an interval of 2.2 months to 37.3 months between cases from the same farm.

**Fig. 1:**
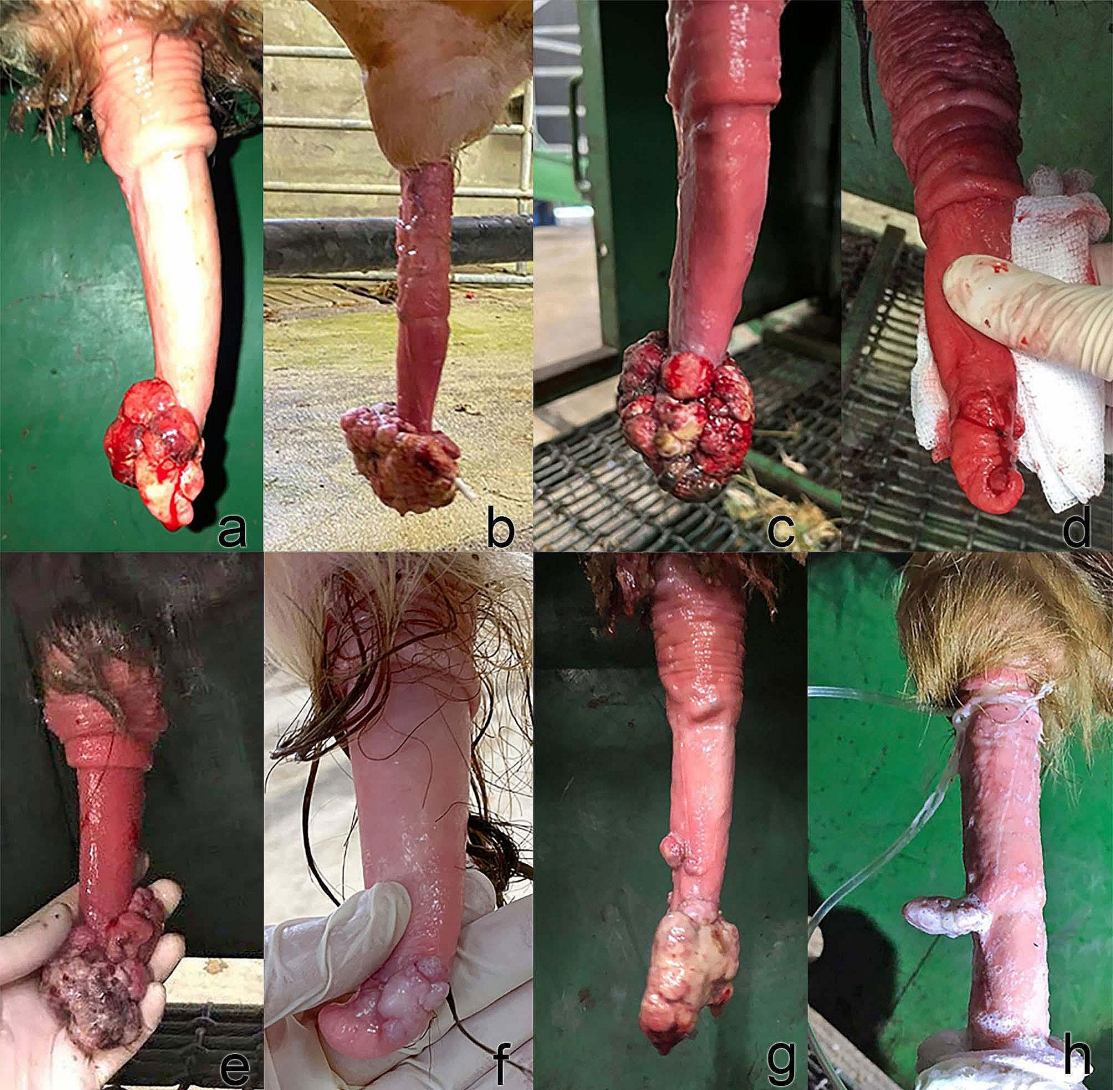
Examples of size and location of various penile fibropapillomas seen over the course of the study; **a-f** show Penile fibropapillomas of the glans penis, **g** shows Penile fibropapillomas of the glans and further proximal and **h** shows a Penile fibropapilloma at the level of the preputial reflection

The clinical records from each case were examined, including photographs from 18 of the 31 cases. The location of the fibropapilloma(s) on the penis varied but, of those evaluated from photographic records, most (14 cases; 78%) were found exclusively on the glans penis (tip), with 3 warts found on the glans plus further proximal on the free portion of the penis (17%), and one at the preputial reflection (5%) (see Fig. 1). Cases of penile fibropapilloma surrounding the glans penis often also involve the urethral serosa (approximately 35% of these cases). This increases the surgical difficulty and may result in unavoidable exposure of the urethral lumen (anecdotally in 30% of cases involving the urethral serosa) during surgical removal necessitating urethral closure. Of the 31 bulls; 28 received antibiotics post-surgery and in the majority (89%) of cases, amoxicillin was used. Only one bull, according to the records, was not given non-steroidal anti-inflammatories pre-operatively.

### Regional anaesthetic approach: xylazine-procaine epidural and internal pudendal nerve block

**Fig. 2:**
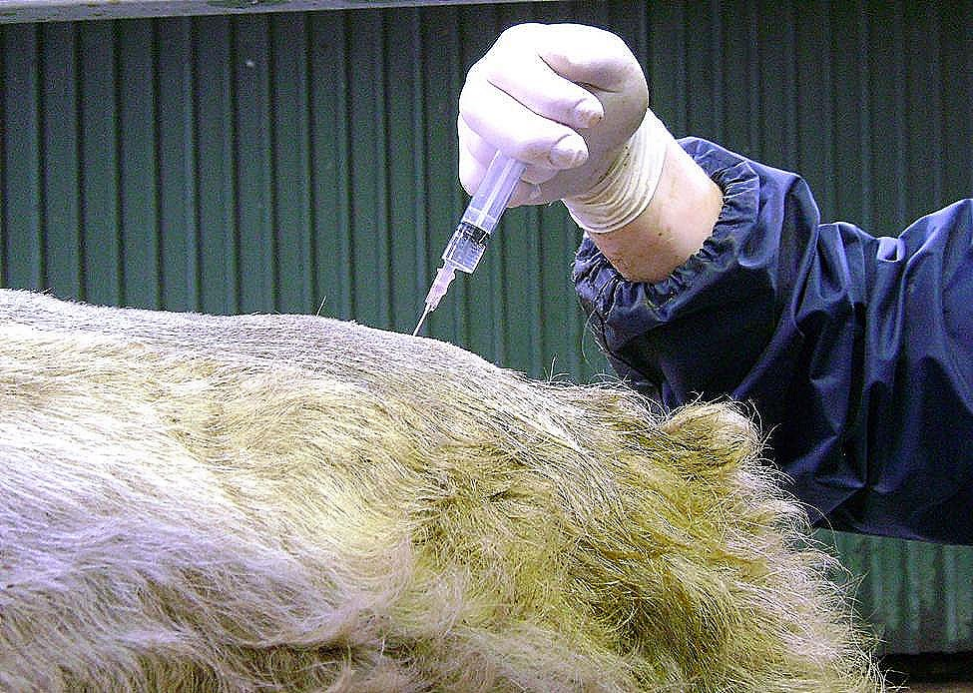
Image showing administration of low volume caudal epidural

Sedation, while not essential, is preferred and recommended by the authors when preparing a bull for surgical removal of penile fibropapillomas using a standing surgical procedure as described later in this case series. The regional anaesthetic approach used in UCDVH was a standing approach which can be carried out in a standard crush with a headgate. It included a low volume caudal xylazine-procaine hydrochloride combination epidural containing 0.03 to 0.07 mg/kg xylazine 2% (Xylapan 2%, Vetoquinol Ireland Limited, 12 Northbrook Road, Ranelagh, Dublin 6, Ireland); this equates to 0.5 to 1 ml xylazine 2% per 300 kg bodyweight, with the remainder of the epidural made up of procaine hydrochloride local anaesthetic (Pronestesic 40 mg/ml, FATRO S.p.A. Via Emilia, 285–40,064, Ozzano Emilia, Bologna, Italy) to the maximum volume of 1 ml per 100 kg bodyweight. This xylazine-procaine epidural provides analgesia of the caudal pelvic region and the skin of the ischiorectal fossae either side of the tailhead, while simultaneously providing mild standing sedation for 40 to 60 min. The first intercoccygeal space was clipped and aseptically prepared and the epidural administered slowly using a syringe and an 18G 1 ½ inch needle (see Fig. 2). While both xylazine 2% and procaine hydrochloride are licensed for use in cattle, and the performance of a xylazine-local anaesthetic epidural is a recognised and documented technique for regional anaesthesia in cattle [18–20], neither of these products are licenced in Ireland for use together as part of a low volume caudal epidural. It is the authors view, however, that the use of these, or similar products, for this regional anaesthetic approach is governed and justifiable under Article 113 on “Use of medicinal products outside the terms of the marketing authorisation in food-producing terrestrial animal species” of Regulation (EU) 2019/6 on veterinary medicinal products.

**Fig. 3:**
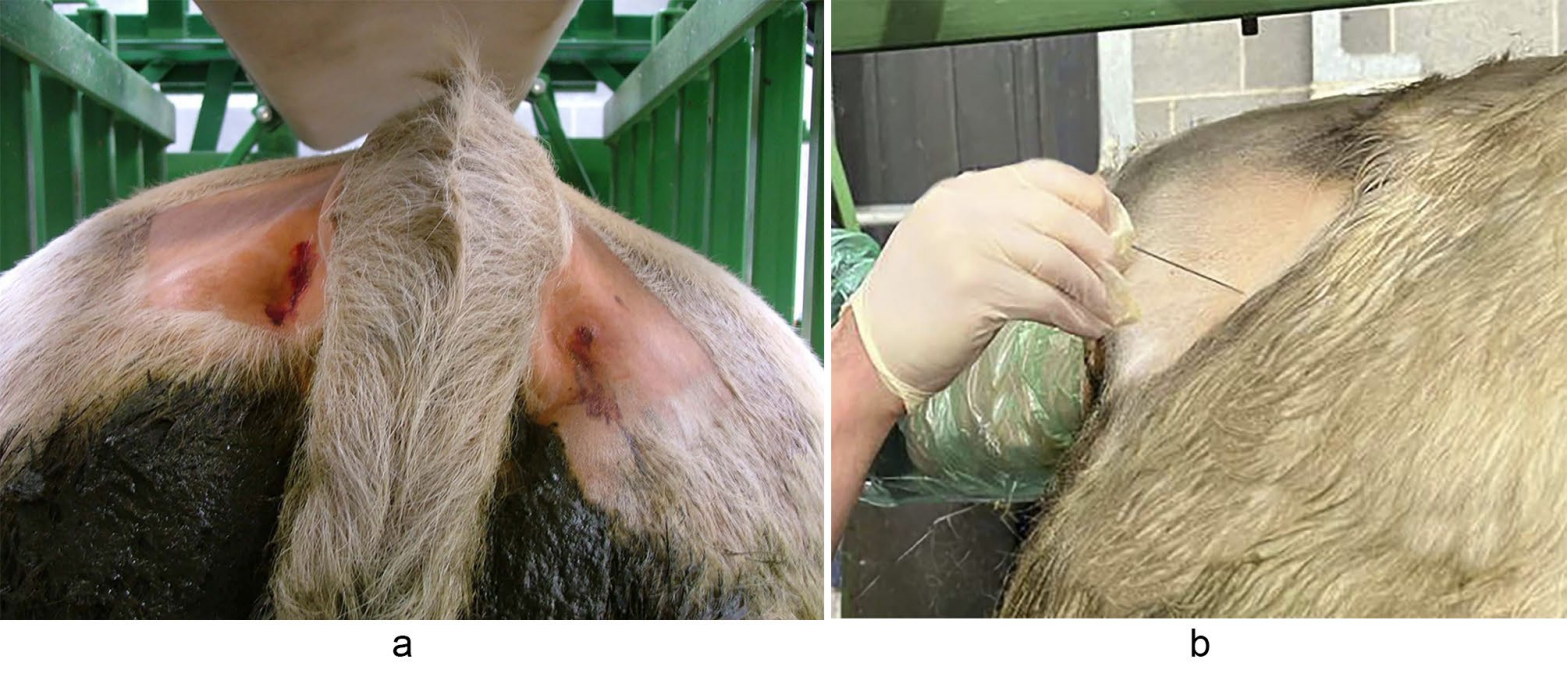
**a**. Image of the correct anatomical site for the internal pudendal nerve block approach. **b**. Image showing appropriate placement of the spinal needle for the internal pudendal nerve block

Both ischiorectal fossae beside the tailhead were clipped and aseptically prepared (see Fig. 3a). A gloved and lubricated hand was placed in the rectum and the sacrosciatic notch located. Anatomically, the internal pudendal nerve, and blood vessels, are located deep to this notch which is approximately 1 cm in diameter [19]. The sacrosciatic notch is found approximately a hands length (15–20 cm) into the rectum on the lateral wall of the pelvis and approximately 4–5 cm dorsal to the pelvic floor in most bulls. A 9 cm 18G spinal needle was placed through the skin of the ischiorectal fossa and advanced at a downward 30-45^o^ angle towards the sacrosciatic notch. The alternate hand placed per rectum was used to feel the location of the spinal needle, ensuring that it did not penetrate the rectal wall, and to guide it towards the sacrosciatic notch (see Fig. 3b). In large (> 700 kg) bulls, it is unlikely that the needle will advance all the way to the notch and is commonly 1.5 to 2 cm short of the notch. Local anaesthetic (procaine hydrochloride) was administered to desensitise the main branch of the internal pudendal nerve, with an increased volume required if there is a greater distance between the tip of the needle and the sacrosciatic notch. Typically, 40 ml of procaine was injected towards the notch, with a slight dorsal to ventral fanning of the syringe and needle. This was followed by withdrawal of the spinal needle by 2–3 cm and redirection inwards and dorsally to allow for infiltration of a further 15-20 ml towards the dorsal branch of the internal pudendal nerve. The same procedure was repeated on the opposite side; including swapping the hands per rectum. Extrusion of the penis is usually seen within 10 to 15 min.

### Surgical procedure: surgical removal of fibropapillomas and cautery

**Fig. 4:**
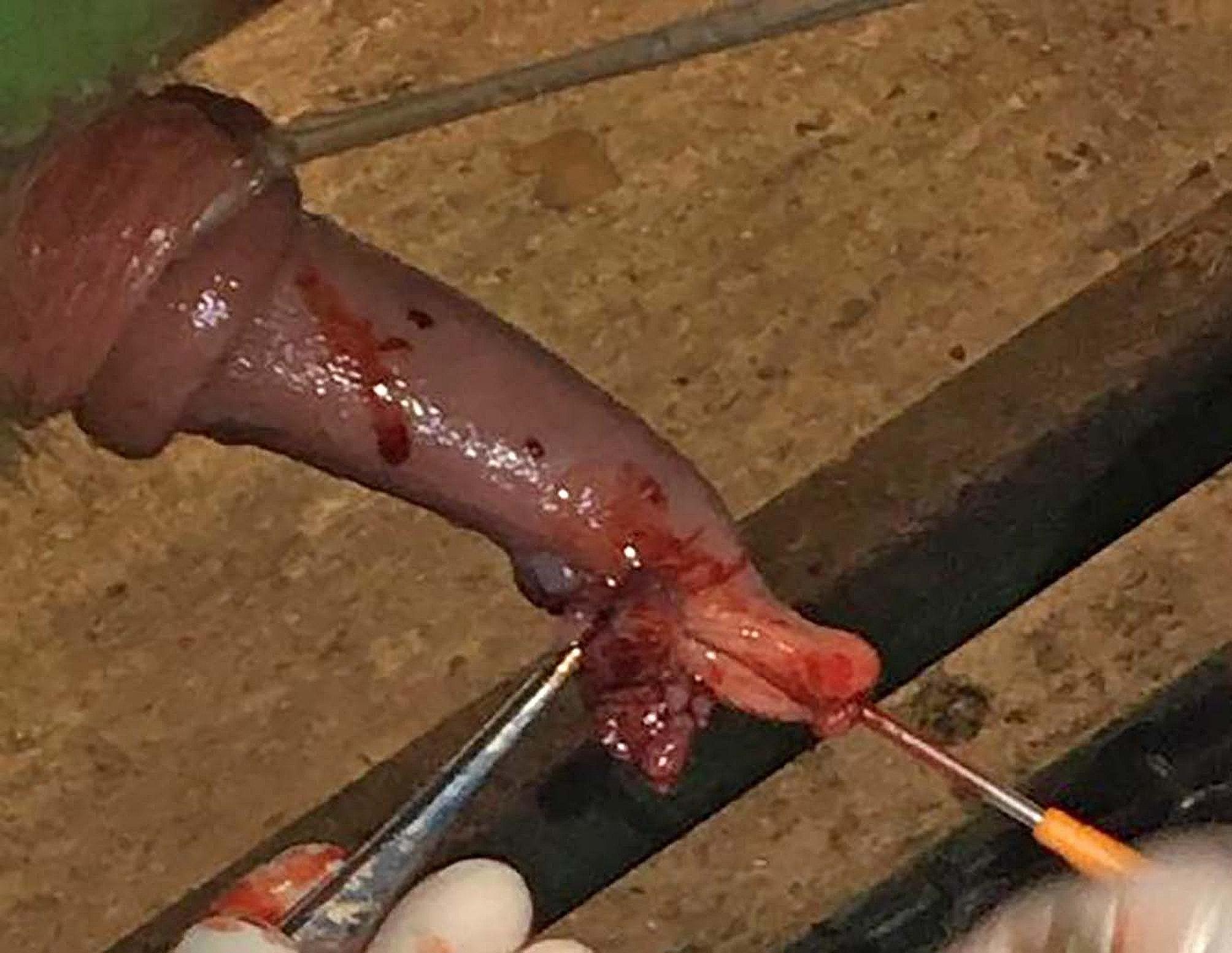
Image showing placement of the tourniquet and urethral catheter prior to surgical excision

Pre-operative medical management in this case series of bulls included 0.5 mg/kg meloxicam (Loxicom 20 mg/ml, Norbrook Laboratories (Ireland) Limited, Rossmore Industrial Estate 6, Monaghan, Ireland) administered subcutaneously. Once the penis had prolapsed from the prepuce and was hanging downwards, the efficacy of the internal pudendal nerve block was tested by pricking the penile serosa with a small 18–20 gauge needle – care must be taken in the standing bull when checking the regional block. A urinary catheter (8-10FG) was inserted into the urethra; this helped with visualisation and repair if the urethra was opened during surgical removal of the fibropapillomas. It is essential to place a tourniquet at the base of the penis, just below the prepuce (see Fig. 4).

**Fig. 5:**
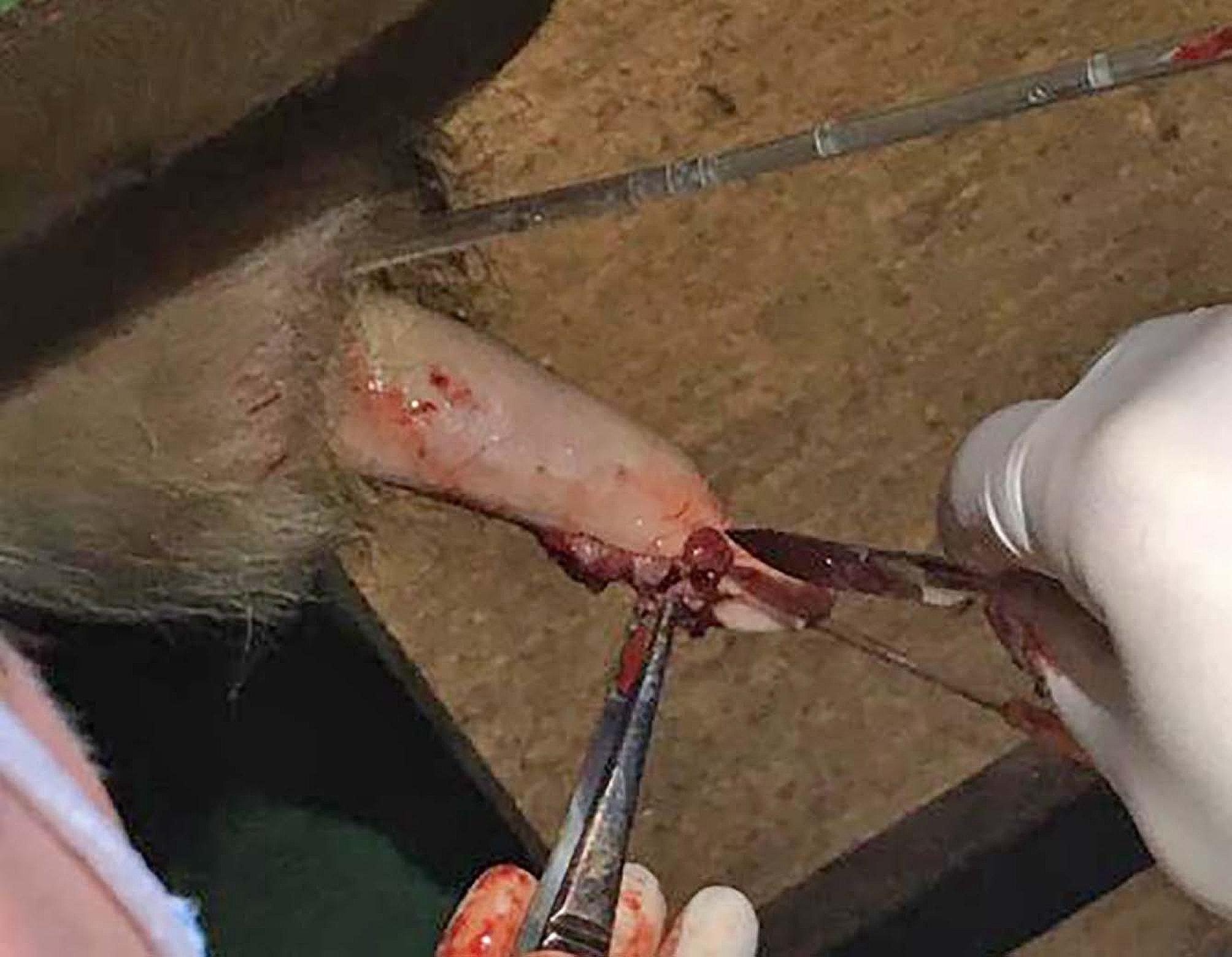
Image showing surgical resection of wart tissue

Dilute chlorhexidine (4% Hibiscrub) was used to clean the penis, with particular attention given to the glans/tip and wart tissue. A size 24 scalpel blade was used to debulk the warts until the stalk/base could be seen (see Fig. 5). Fibropapillomas frequently invade through the full thickness of the penile serosa requiring deep removal and exposure of the submucosa.

**Fig. 6:**
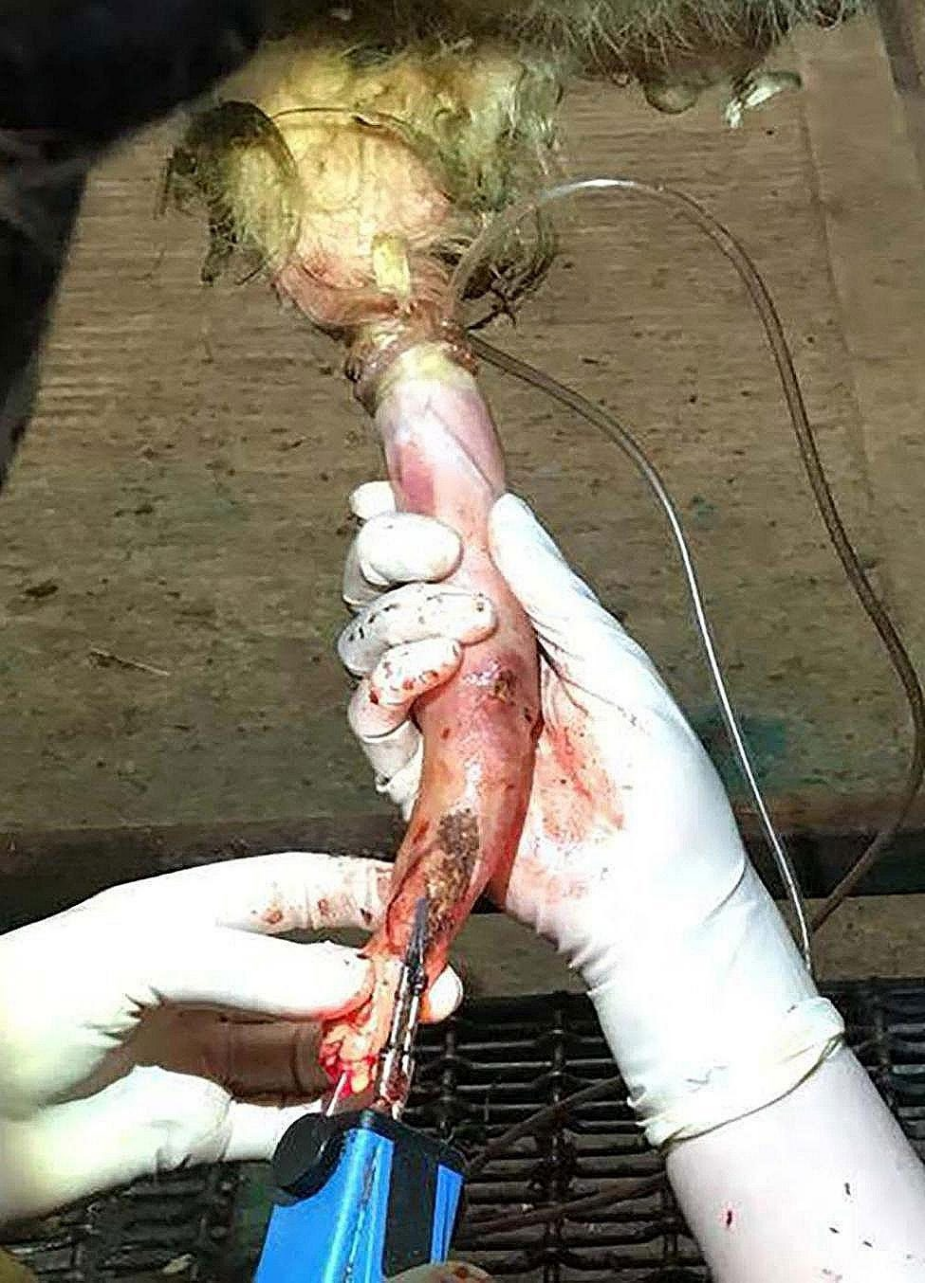
Image showing cautery of penile submucosa at the glans/tip of the penis

**Fig. 7:**
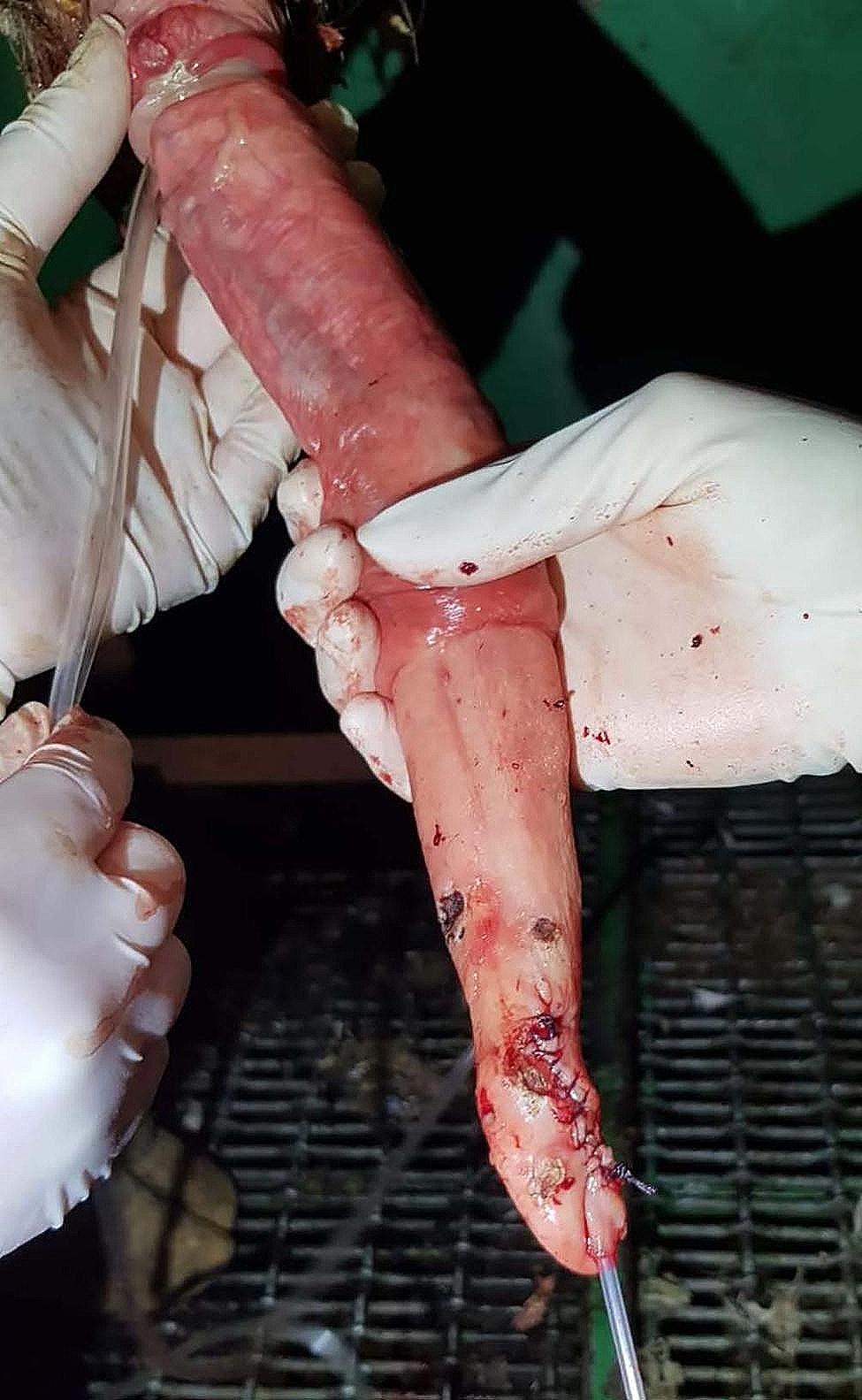
Image showing the modified utrecht suture pattern after iatrogenic urethral incision

Cautery was used for haemostasis (see Fig. 6). Initial primary cauterisation was carried out following fibropapilloma removal and prior to the removal of the tourniquet. In cases where surgical resection necessitated incision of the penile serosa along the penile shaft closer to the prepuce, it was possible to suture the serosa using 2 − 0 PDS or Vicryl with a round-bodied needle as it was more loose further from the glans penis. However, the majority of fibropapillomas surrounded the glans/tip of the penis where the submucosa was usually exposed during complete wart removal and it was preferable to concentrate on cautery. Additionally, removal of fibropapillomas adhered to the external urethral wall sometimes led to iatrogenic incision through the urethral wall exposing the previously placed urethral catheter. If opened, the urethra was closed using 3 − 0 PDS in an inverting modified Utrecht or Cushings pattern and oversewn (see Fig. 7).

**Fig. 8:**
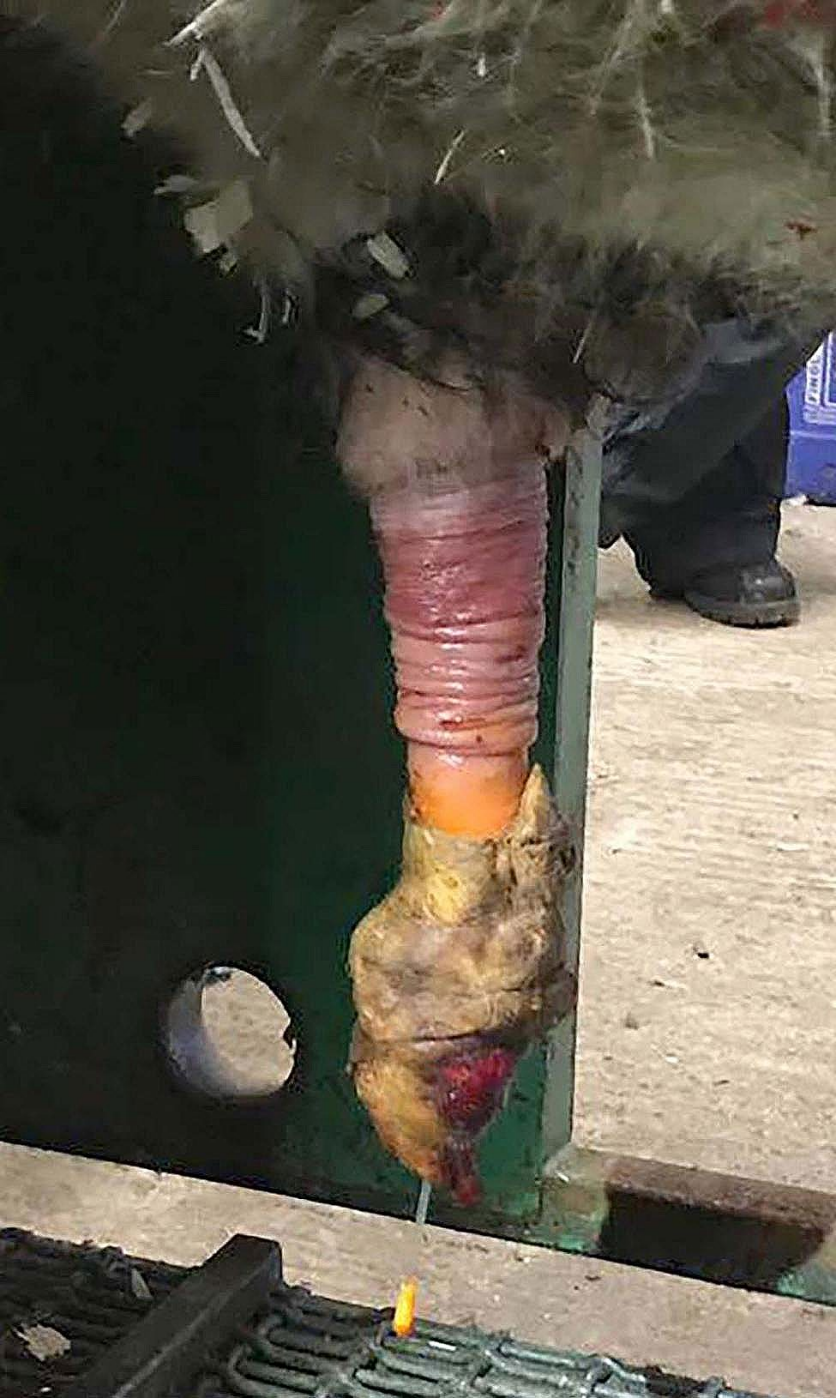
Image showing application of iodine-soaked cotton wool for haemostasis post-operatively

**Fig. 9:**
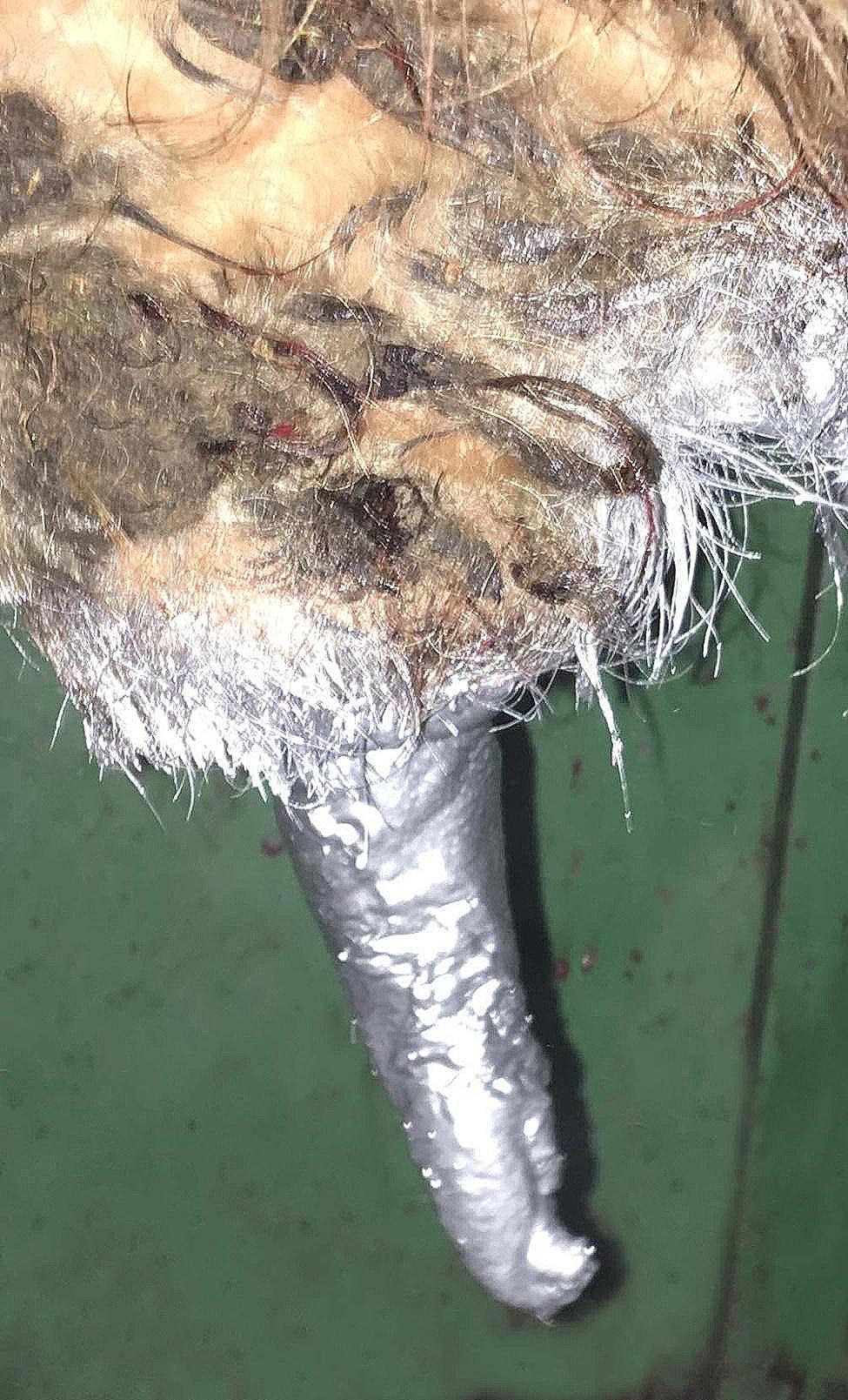
Image showing application of aluminium spray post-operatively

Following cauterisation of exposed submucosa, the tourniquet was removed and further cautery carried out as appropriate. Final capillary bleeding was stopped by placing iodine-soaked cotton wool around the penis. This helped with haemostasis and post-operative cleaning. Cotton wool was placed around the surgical area and left hanging on the penis for 2 to 3 min (see Fig. 8) and, if capillary bleeding had stopped after removal of the cotton wool, the penis was sprayed with aluminium spray (Aluspray, Vetoquinol Ireland Limited, 12 Northbrook Road, Ranelagh, Dublin 6, Ireland) as a topical barrier spray, and aid to wound healing [21], prior to removal of the urinary catheter (see Fig. 9). Administration of 15 mg/kg amoxicillin (Betamox LA 150 mg/ml, Norbrook Laboratories Limited, Station Works, Camlough Road, Newry, Co. Down, BT35 6JP, United Kingdom) intramuscularly was typically used for antibiosis post-operatively.

Discharge instructions pertaining to each bull issued to the farmers were to allow at least three weeks of sexual rest following the procedure. Farmers were also advised to keep bulls separate in clean, well-bedded straw pens.

## Follow-up

Phone calls were made to each of the 15 farmers between May and June 2023, after their initial visit to UCDVH. Of these, three farmers were not able to be contacted despite two attempts. Each farmer was asked if their bull had experienced a recurrence of penile fibropapillomas after their surgery at UCDVH. However, no other questions were asked regarding the breeding soundness or future fertility of the bulls. Some of these bulls were not retained by the original farmers and had been sold on to farmers unknown to the hospital. Out of the 28 cases where the owners were able to be contacted, only two reported recurrence of the warts. This equates to a 7.1% recurrence rate, which is lower than that quoted in the literature. Historically, the recurrence rate was thought to be 10%; with lesions being obvious at 3 to 4 weeks post-surgery [22]. Of these two cases of recurrence, one returned to the hospital for further surgery and the other was treated conservatively at home.

## Discussion and conclusions

In this case series presented to UCDVH, we have shown that surgical resection of penile fibropapillomas was mainly performed on bulls under the age of two years old. The oldest bull that was treated was 27.8 months old, which is consistent with the literature that suggests fibropapillomas are generally found in bulls younger than two years of age [1] and rarely persist beyond three years of age [22]. It was also found that 53% of farms experienced more than one case. This finding may corroborate the suspected transmission methods of the virus; namely homosexual behaviour in a group of young bulls [10] and multiple cases [11] within a close contact group [4]. Almost half of the cases seen at UCDVH were in Hereford bulls, and another study has found that Herefords were over-represented when looking at penile and preputial abnormalities [23]. The majority of the lesions that were documented with photographs (18/31 cases) were located on the glans/tip of the penis (78%), which also corresponds with information reported in the literature [4, 24].

The use of the internal pudendal nerve block, discussed in other papers [4, 25], allowed for standing surgery to be performed compared to local anaesthesia of the penile mucosa which may require the bull to be sedated and placed in lateral recumbency [9, 24] or full general anaesthesia with the accompanying risk of anaesthetic-induced complications e.g. bloat, regurgitation, hypotension, hypoxia and increased risk of mortality [26]. Surgical removal with cauterisation has proven to be an effective treatment method, with 7.1% of cases recurring in this case series, compared to 10% quoted in the literature [15]. The surgical procedure carried out in UCDVH involving deep removal of the fibropapillomatous tissue exposing the healthy submucosa, followed by extensive cauterisation, may explain the low recurrence rate. Histologically it has been shown that there are finger-like projections from the epidermis into the dermis in these warts [9], suggesting that inflammatory cells could be left behind if only the superficial part of the fibropapilloma is excised. Anecdotal evidence exists that bulls are able to pass a BBSE as early as two weeks following surgical resection of penile fibropapillomas using the technique described in this case series. Other surgical techniques including excision or electrocautery [24] and cryotherapy [27, 28] have been described and may also be efficacious in destroying the abnormal epidermal cells.

There are two alternative non-surgical treatment options also: (i) allowing time for spontaneous regression [7] or (ii) administration of an autogenous vaccine [29]. However, it was reported that an autogenous vaccine could be used preventatively but was not curative of existing warts [4] and that it did not result in regression any quicker than spontaneous regression [30].

Limitations of this case series include missing data points and photographs due to the retrospective nature of the study. Additionally, there was a protracted follow-up period and reliance on owner response to determine recurrence rate which is not as reliable as a prospective approach. No further diagnostics were performed other than the initial clinical examination; histopathology could be used in the future to confirm the diagnosis and the complete excision of the fibropapilloma. Assessment of fertility after surgery would be an interesting addition in future studies. In conclusion, this is the first case series that the authors are aware of describing cases of penile fibropapillomatosis in bulls and describing the regional anaesthetic and surgical techniques in detail. The surgical management described can be used in the field on a standing animal and the results; including the age of affected bulls and location of the fibropapillomas, confirm much of the scientific data available on penile fibropapillomas.

## Data Availability

The datasets used and/or analysed during the current study are available from the corresponding author on reasonable request.

## Abbreviations

BBSE: Bull Breeding Soundness Exam
BPV: Bovine Papilloma Virus
UCDVH: University College Dublin Veterinary Hospital
